# Comparing machine learning algorithms to predict COVID‑19 mortality using a dataset including chest computed tomography severity score data

**DOI:** 10.1038/s41598-023-38133-6

**Published:** 2023-07-13

**Authors:** Seyed Salman Zakariaee, Negar Naderi, Mahdi Ebrahimi, Hadi Kazemi-Arpanahi

**Affiliations:** 1 Department of Medical Physics, Ilam University of Medical Sciences, Ilam, Iran; 2 Department of Midwifery, Ilam University of Medical Sciences, Ilam, Iran; 3 Department of Emergency Medicine, Tehran University of Medical Sciences, Tehran, Iran; 4 Department of Health Information Technology, Abadan University of Medical Sciences, Abadan, Iran

**Keywords:** Infectious diseases, Mathematics and computing, Information technology

## Abstract

Since the beginning of the COVID-19 pandemic, new and non-invasive digital technologies such as artificial intelligence (AI) had been introduced for mortality prediction of COVID-19 patients. The prognostic performances of the machine learning (ML)-based models for predicting clinical outcomes of COVID-19 patients had been mainly evaluated using demographics, risk factors, clinical manifestations, and laboratory results. There is a lack of information about the prognostic role of imaging manifestations in combination with demographics, clinical manifestations, and laboratory predictors. The purpose of the present study is to develop an efficient ML prognostic model based on a more comprehensive dataset including chest CT severity score (CT-SS). Fifty-five primary features in six main classes were retrospectively reviewed for 6854 suspected cases. The independence test of Chi-square was used to determine the most important features in the mortality prediction of COVID-19 patients. The most relevant predictors were used to train and test ML algorithms. The predictive models were developed using eight ML algorithms including the J48 decision tree (J48), support vector machine (SVM), multi-layer perceptron (MLP), k-nearest neighbourhood (k-NN), Naïve Bayes (NB), logistic regression (LR), random forest (RF), and eXtreme gradient boosting (XGBoost). The performances of the predictive models were evaluated using accuracy, precision, sensitivity, specificity, and area under the ROC curve (AUC) metrics. After applying the exclusion criteria, a total of 815 positive RT-PCR patients were the final sample size, where 54.85% of the patients were male and the mean age of the study population was 57.22 ± 16.76 years. The RF algorithm with an accuracy of 97.2%, the sensitivity of 100%, a precision of 94.8%, specificity of 94.5%, F1-score of 97.3%, and AUC of 99.9% had the best performance. Other ML algorithms with AUC ranging from 81.2 to 93.9% had also good prediction performances in predicting COVID-19 mortality. Results showed that timely and accurate risk stratification of COVID-19 patients could be performed using ML-based predictive models fed by routine data. The proposed algorithm with the more comprehensive dataset including CT-SS could efficiently predict the mortality of COVID-19 patients. This could lead to promptly targeting high-risk patients on admission, the optimal use of hospital resources, and an increased probability of survival of patients.

## Introduction

In December 2019, the outbreak of the novel coronavirus disease (COVID‑19) also known as severe acute respiratory syndrome coronavirus 2 (SARS-COV-2) was reported for the first time^1^. This life-threatening infection is caused by a recently originating zoonotic virus named severe acute respiratory syndrome coronavirus 2 (SARS-CoV-2)^2^.

COVID-19 is a highly contagious viral infection and continued to spread aggressively worldwide, despite all the preventive and lockdown measures. Its clinical outcomes ranged from asymptomatic to mild or moderate symptoms, and critical complications or death in some cases^3^. Twenty percent of COVID-19 patients must be hospitalized and, approximately, 20–30% of in-hospital COVID-19 patients need intensive care unit (ICU) care^4^. The pooled case fatality rates (CFR) of in-hospitalized and ICU-admitted patients were 13% (95% CI 9.0–17.0, P < 0.001, I^2^ = 95.6) and 37.0% (95% CI 24.0–51.0, P < 0.001, I^2^ = 97.8), respectively^5^. This complex and highly contagious virus had made a tremendous impact on the health of people all over the world and caused a significant number of deaths, particularly for the elderly, pregnant women, and infected cases with underlying comorbidities such as low immune functions, cardiopulmonary diseases, cancer, infectious diseases, hypertension, and diabetes^6^.

Hence, early identification of patients who are at risk of mortality is necessary to mitigate the burden on the healthcare system and to reduce deaths as much as possible. A predictive model that accurately predicts the poor outcomes for COVID-19 patients could assist in efficiently allocating limited medical resources, improve the quality of health care, and eventually optimize patient management^7,8^.

The disease behavior and courses are unpredictable which made the diagnosis of high-risk patients with poor prognoses a challenging problem^9^. Predictions made by different computational and statistical models are used to respond to this challenge^10,11^.

Since the beginning of the COVID-19 pandemic, new and non-invasive digital technologies such as artificial intelligence (AI) had been introduced for mortality prediction of COVID-19 patients. In AI problems, machines learn from past experiences and would adjust to new inputs. The machine learning (ML) approach as a subfield of AI is a complex and flexible classification modeling that leverages big datasets to reveal significant hidden relationships or patterns^12^. For the prediction of clinical outcomes in COVID-19 patients, ML methods have more accurate results than conventional statistics models^13^. The prognostic performances of the ML-based models for predicting clinical outcomes of COVID-19 patients had been mainly evaluated using demographics, risk factors, clinical manifestations, and laboratory results^14–17^. There is a lack of information about the prognostic role of imaging manifestations in combination with demographics, clinical manifestations, and laboratory predictors. Computed tomography (CT) scan is a valuable method routinely used in the diagnosis, monitoring, and management of COVID-19 patients. A significant correlation between the chest computed tomography severity score (CT-SS), which is determined based on the severity of pulmonary involvement on CT scans, and mortality in COVID-19 patients has been reported^1,18,19^. This pulmonary involvement score was proposed as an appropriate prognostic factor for mortality prediction in COVID-19 patients by recent meta-analysis studies^1,19^. Thus, CT-SS might improve the prognostic performances of the ML algorithms for mortality prediction of COVID-19 patients.

The purpose of the present study is to develop an efficient ML prognostic model for mortality prediction of COVID-19 patients based on a more comprehensive dataset including CT-SS, demographics, clinical manifestations, and laboratory predictors. Therefore, this study seeks to answer two questions. What are the most relevant predictors of patients’ mortality? And using the most relevant predictors of patients’ mortality, which ML model is more effective for mortality prediction of COVID-19 patients?

## Methods

### Dataset description

In this study, a COVID-19 hospital-based registry database was retrospectively reviewed from February 9, 2020, to December 20, 2020. This dataset included the data of the patients referred to Ayatollah Talleghani Hospital (COVID-19 referral centre), Abadan city, Iran.

A total of 6854 suspected cases had been referred to the hospital’s ambulatory and emergency departments (EDs), of whom 1853 cases were introduced as positive RT-PCR COVID-19, 2472 as negative, and 2529 as unspecified.

In the COVID-19 hospital-based registry database, seventy-two primary features in six main classes including patient’s demographics (eight features), clinical features (21 features), history of personal diseases/comorbidity (13 features), laboratory results (28 features), CT-SS (one feature), and an output variable (0: survived and 1: deceased) had been registered for COVID-19 patients. Primary features registered in the COVID-19 hospital-based registry database are listed in Table 1. Numerical parameters were quantitatively measured and nominal parameters were registered as Yes or No. In this database, demographic information of patients and their history of personal diseases/comorbidity were registered from the medical records or by asking the patient and the patient's companions. For each patient, the clinical features including cough, fever, shortness of breath, loss of smell, loss of taste, etc. were registered at the time of admission. In the first 24 h hospitalization of the patients, their blood and urine samples were analyzed and they were subjected to chest CT imaging. The laboratory results were automatically registered in their medical records.

**Table 1:** Primary features registered in the COVID-19 hospital-based registry database.

No.	Features name	Variable type	No.	Features name	Variable type
Demographics			37	Autoimmune disease	Nominal
1	Age	Numeric	38	Liver disease	Nominal
2	Height	Numeric	39	Metabolic diseases	Nominal
3	Weight	Numeric	40	Neurological disorders	Nominal
4	Sex	Nominal	41	Kidney disease	Nominal
5	Marital status	Nominal	42	Cancer	Nominal
6	Smoking	Nominal	Laboratory results		
7	Drug addiction	Nominal	43	Serum creatinine	Numeric
8	Alcohol consumption	Nominal	44	Red-cell count	Numeric
Clinical features			45	White-cell count	Numeric
9	ARDS	Nominal	46	Haematocrit	Numeric
10	ARDS confirmed by X-ray examinations	Nominal	47	Haemoglobin	Numeric
11	Arthralgia	Nominal	48	Platelet count	Numeric
12	Cough	Nominal	49	Absolute lymphocyte count	Numeric
13	Conjunctivitis	Nominal	50	Absolute neutrophil count	Numeric
14	Contusion	Nominal	51	Calcium	Numeric
15	Nausea	Nominal	52	Phosphorus	Numeric
16	Vomit	Nominal	53	Magnesium	Numeric
17	Headache	Nominal	54	Sodium	Numeric
18	Muscular pain	Nominal	55	Potassium	Numeric
19	Chill	Nominal	56	Blood urea nitrogen	Numeric
20	Fever	Nominal	57	Total bilirubin	Numeric
21	Diarrhoea	Nominal	58	Aspartate aminotransferase	Numeric
22	Pneumonia	Nominal	59	Alanine aminotransferase	Numeric
23	Pneumonia confirmed by X-ray examinations	Nominal	60	Albumin	Numeric
24	Lung inflammation	Nominal	61	Glucose	Numeric
25	Runny nose	Nominal	62	Lactate dehydrogenase	Numeric
26	Sore throat	Nominal	63	Creatine kinase	Numeric
27	Shortness of breath	Nominal	64	Activated partial thromboplastin time	Numeric
28	Loss of smell	Nominal	65	Prothrombin time	Numeric
29	Loss of taste	Nominal	66	Alkaline phosphatase	Numeric
History of personal diseases/comorbidity			67	C-reactive protein	Numeric
30	Underlying disease	Nominal	68	Erythrocyte sedimentation rate	Numeric
31	Heart disease	Nominal	69	D-dimer	Numeric
32	Hypertension	Nominal	70	Hypersensitive troponin	Numeric
33	Chronic lung disease	Nominal	Imaging results		
34	Diabetes	Nominal	71	CT-SS	Numeric
35	Dialysis	Nominal	Output		
36	Hemoglobinopathy disease	Nominal	72	Mortality	Nominal

Chest CT scores quantify the severity of pulmonary involvement in CT images. For each patient, five lung lobes were visually scored as 0 (no involvement), 1 (less than 5% involvement), 2 (5–25% involvement), 3 (25–50% involvement), 4 (50–75% involvement), and 5 (75–100% involvement). The total CT-SS is the sum of the individual lobar scores and ranges from 0 to 25. All CT images were separately reviewed by two radiologists. Any disagreements were resolved through consulting with an attending radiologist with 23 years of experience.

### Data pre-processing

Data pre-processing is an imperative step to address irrelevant, redundant, and unreliable data and it could significantly resolve inconsistencies^20^. In this paper, data pre-processing was performed before the training of the ML models. First, records with more than 70% of missing data were excluded from the dataset. The remaining missing values of continuous and discrete variables were imputed by mean and mode values, respectively. Noisy and abnormal values, errors, and meaningless data were addressed by an expert panel including one health information management expert (HKA), two infectious diseases specialists, and two haematologists.

**Figure 1:**
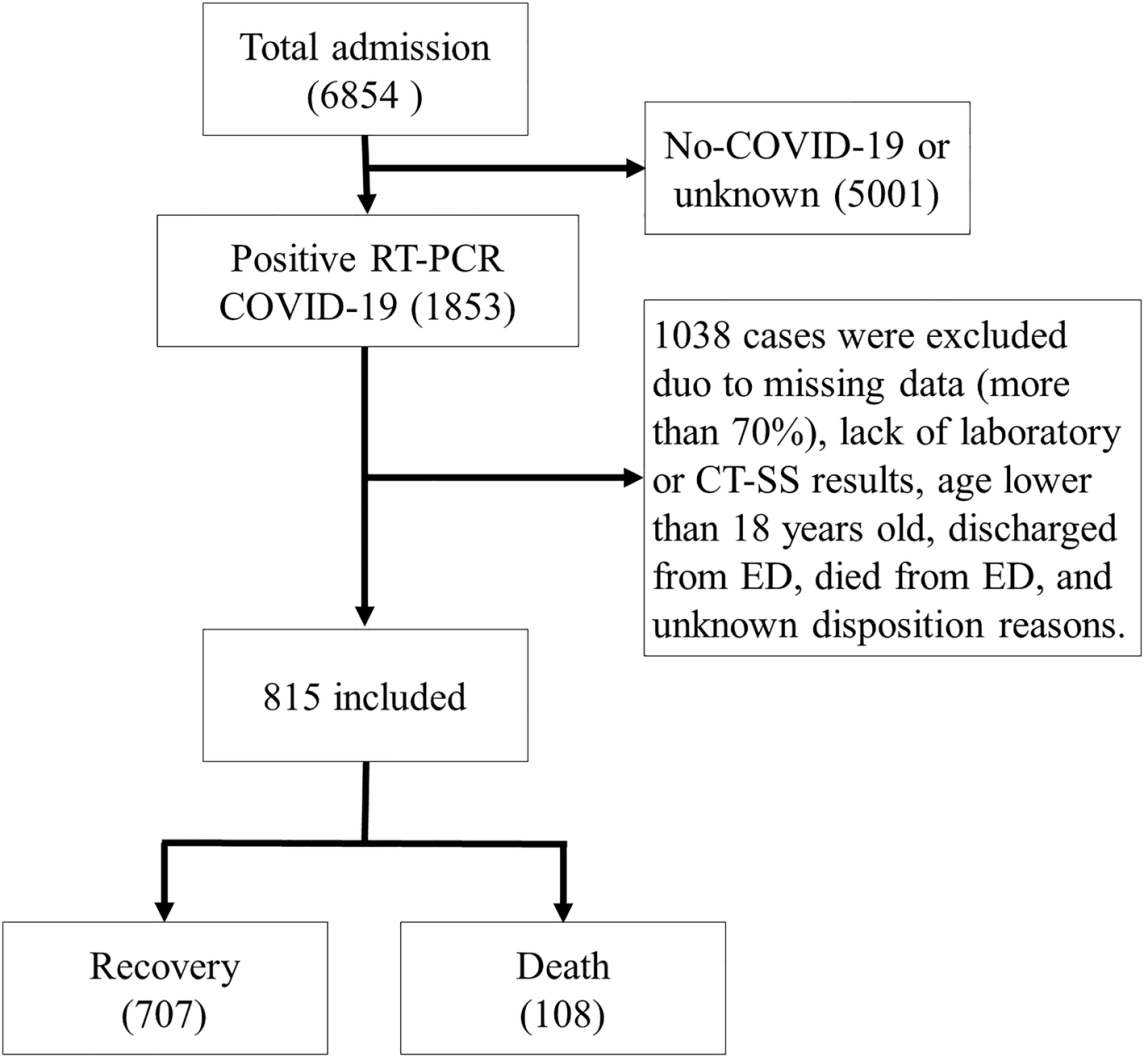
Flow chart describing patient selection.

The positive RT-PCR COVID-19 cases were only entered into the study. Negative RT-PCR COVID-19 test, unknown dispositions, discharge or death from the emergency department, missing data > 70%, and age lower than 18 years old were the study exclusion criteria. Figure 1 depicted the schematic of the study inclusion and exclusion criteria. After applying the inclusion/exclusion criteria, the final sample size was 815 patients.

This dataset contains 707 and 108 cases in the survival and death groups, respectively. This imbalanced input would cause delivering biased results toward the dominant class.

The problem of the imbalanced dataset was dealt with using the synthetic minority over-sampling technique (SMOTE) method (https://imbalanced-learn.org/stable/). SMOTE algorithm is the most frequently employed synthetic oversampling which creates synthetic samples of the minority class using randomly selected instances of the minority class and their k nearest neighbors^21^. In this method, a random data instance with its k nearest neighbors are selected. Then, the second data instance would be selected from the k nearest neighbors set. The new synthetic sample is generated along the line joining the two samples as a convex combination. This procedure would be repeated until the minority class is balanced with the majority one^22^. Unlike the random oversampling method, the risk of overfitting was avoided in SMOTE method and this method can yield relatively better results^23^.

### Feature selection

**Figure 2:**
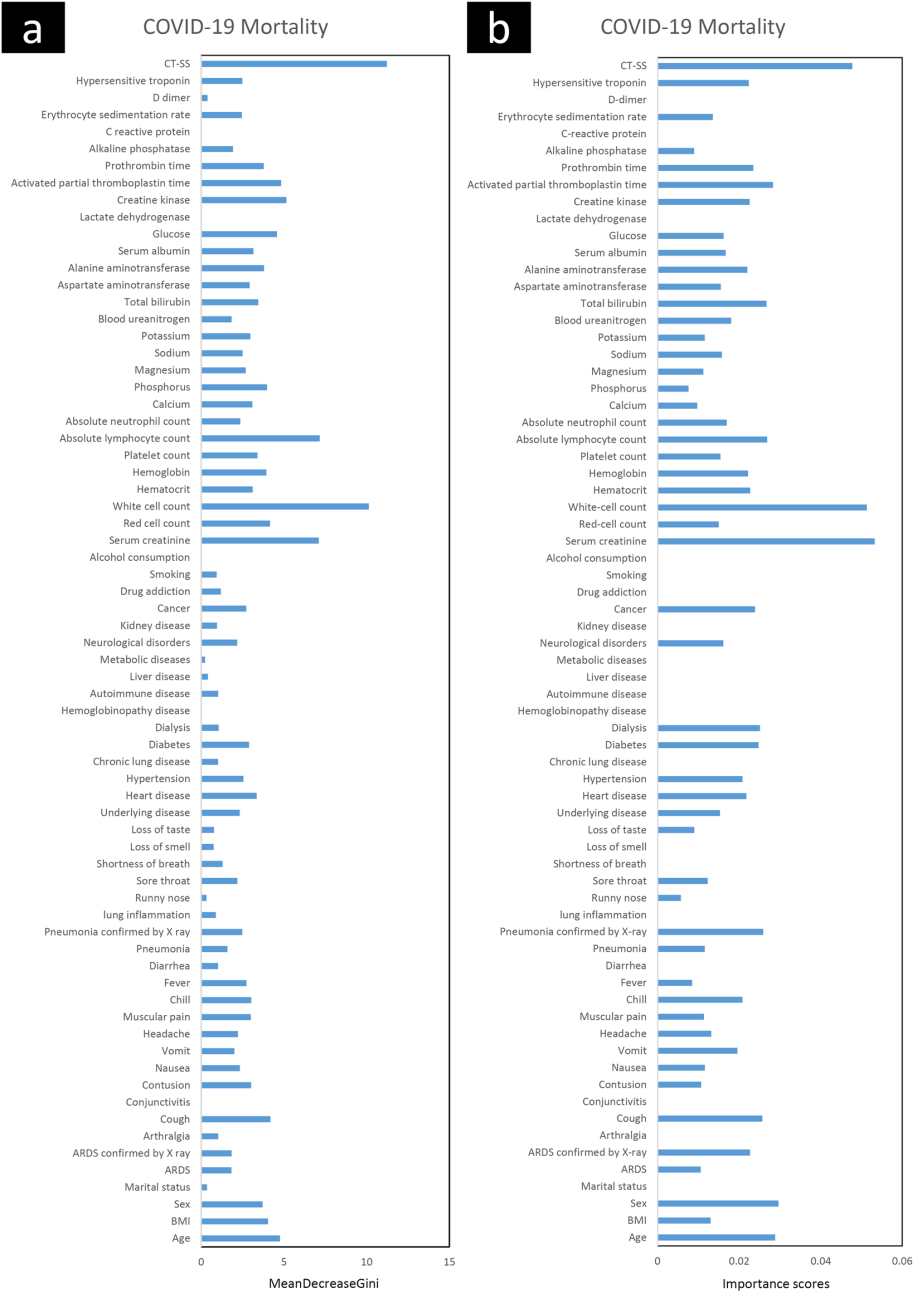
The importance scores of the predictors calculated using random forest (**a**) and XGBoost (**b**) tests.

The feature selection process is widely used in data mining to determine the most important variables highly correlated with the output variable^24^. One of the main advantages of using this method is to prevent overfitting of the ML algorithms^25^. In this study, the most important variables for mortality prediction of COVID-19 were determined using XGBoost, random forest, and Chi-squared tests. The chi-squared test evaluates the statistical differences in the parameters between the deceased and survived groups. The importance scores of the predictors calculated using XGBoost and random forest tests are depicted in Fig. 2. In all feature selection methods, a high score was achieved for strong predictors such as CT-SS, WBC, serum creatinine, etc. But, there were significant discrepancies in the importance scores calculated using XGBoost and random forest tests for some parameters. The dialysis history of the patient has moderate importance in the XGBoost method and the random forest algorithm assigned low importance to it. There was no statistically significant difference in the dialysis history of the patient between the deceased and survived cases (P = 0.011). In another hand, phosphorus concentration in blood samples has low and moderate importance scores in XGBoost and random forest methods, respectively. A strong predictor must first have a statistically significant difference between the deceased and survived cases in order to predict the mortality of COVID-19 patients correctly. According to the observed discrepancies and to determine the predictors which have significant differences between the deceased and survived cases, the independence test of Chi-square was used to determine the most important variables in the mortality prediction of COVID-19 patients. The predictors selected by the Chi-square test had moderate to high importance scores in XGBoost and random forest methods. It is worth mentioning that predictors such as CT-SS have high importance scores in both XGBoost and random forest tests and they have a significant statistical difference between the deceased and survived cases (P < 0.001). The SPSS software (version 23) was used to calculate the Chi-square coefficients and P < 0.01 was regarded as the significant level.

### Model development

In this study, the predictive models were developed using eight ML algorithms including the J48 decision tree (J48), support vector machine (SVM), multi-layer perceptron (MLP), k-nearest neighbourhood (k-NN), Naïve Bayes (NB), logistic regression (LR), random forest (RF), and eXtreme gradient boosting (XGBoost)^22^. These mortality prediction models were implemented using Waikato Environment for Knowledge Analysis (Weka) software (version 3.9.2, University of Waikato, New Zealand). The k-fold cross-validation method was used in the performance evaluation of the developed classifiers. The k-fold cross-validation method has a relatively low level of bias and variation, which makes it a preferred technique. The parameters of the selected ML algorithms for COVID-19 mortality prediction are described in Table 2.

**Table 2:** The parameters of the selected ML algorithms for COVID-19 mortality prediction.

ML algorithm	Parameter
J48	Batch size^a^ = 100, the confidence factor used for pruning = 0.25, the minimum number of instances per leaf = 2, number of folds^b^ = 3, unpruned decision tree = False
NB	Batch size = 100
LR	Batch size = 100, ridge value in the log-likelihood = 1E-8, number of iterations to perform = 1
MLP	Batch size = 100, The learning rate for weight updates = 0.3, momentum applied to the weight updates = 0.2, validation threshold used to terminate validation testing = 20
SVM	Batch size = 100, the epsilon for round-off error = 1E-12, kernel function = poly kernel-E 1-C 250,007, number of folds = 1, tolerance parameter = 0.001
k-NN	Batch size = 100, K = 29, distance weighting = weight by 1/distance, nearest neighbor search algorithm = linear NN search
RF	Size of each bag, as a percentage of the training set size = 100, batch size = 100, The number of execution slots to use for constructing the ensemble = 1, the number of trees in the random forest = 100
XGBoost	Gamma = 0.1, maximum depth = 0.5, lambda = 3.0, silent = 1.0, eta = 0.1, number of iterations = 100

J48: J48 decision tree; NB: Naïve Bayes; LR: logistic regression; MLP: multi-layer perceptron; SVM: support vector machine; k-NN: k-nearest neighbourhood; RF: random forest; XGBoost: eXtreme gradient boosting.

^a^Batch size is the preferred number of instances to process if batch prediction is being performed.

^b^Number of folds determines the amount of data used for reduced-error pruning.

The performances of the predictive models were evaluated using accuracy, precision, sensitivity, specificity, and area under the ROC curve (AUC) metrics. These performance metrics were compared for all ML algorithms to determine the best model for mortality prediction of COVID-19 patients.

### Ethical considerations

The ethical committee board of Abadan University of Medical Sciences approved the study (Ethics code: IR.ABADANUMS.REC.1401.124). To protect the privacy and confidentiality of patients, the unique identification information of patients was concealed during all steps of the study. All methods of the present study were performed in accordance with the relevant guidelines and regulations. Informed consent was obtained from all subjects and/or their legal guardian(s).

## Results

A total of 6854 suspected cases had been referred to Ayatollah Talleghani Hospital, where records of 815 positive RT-PCR patients remained after applying the exclusion criteria. Overall, 54.85% of the enrolled patients were male and the mean age of the study population was 57.22 ± 16.76 years. As was mentioned, the deceased group contained only 108 records (13%) and SMOTE method has been used to balance these data. The number of records in this class was raised to 707 after balancing the dataset.

### Feature selection

Twenty-seven features were chosen as the most important and relevant predictors using the independence test of Chi-square. These features included demographics, risk factors, clinical manifestations, laboratory results, and CT-SS data. The list of the most important variables and results of the independence test of Chi-square are demonstrated in Table 3. In this table, the mean decreases in Gini and the importance scores of these variables, calculated using XGBoost and random forest tests, were also listed. Descriptive statistics of these features are listed in Table 4. In this study, in agreement with other studies that have reported some important clinical predictors for COVID-19 patient mortality, the most relevant features included age^6,11,15–17,26–30^, gender^6,16,17,26,29–34^, dry cough^6,11,14,17,27,29,32,33,35^ as the clinical symptom, underlying diseases including cardiovascular disease^6,15,17,27,28,34,36,37^, hypertension^6,15,17,27,29,30,34,36^, diabetes^6,15–17^, neurological disease^6,16,17^, cancer^6,17,26,29,37^, laboratory indices such as serum creatinine^6,17^, RBC^6^, WBC^6,29,35^, haematocrit^6^, absolute lymphocyte count^6,14,17,27,31,33,34^, absolute neutrophil count^6,14,15,17,27,28,33,35,36^, calcium^6,11,33^, phosphor^6^, blood urea nitrogen^6,14,33^, total bilirubin^6,35^, serum albumin^6,14,29,33,34^, glucose^6,17^, creatinine kinase^6,11,14,29,34,35^, activated partial thromboplastic time^6^, prothrombin time^6,34^, hypersensitive troponin^6,17,28^, and CT-SS as the imaging manifestation^6,28^. These predictors were used as inputs to develop ML-based models for mortality prediction of COVID-19 patients.

**Table 3:** The importance scores, the mean decreases in Gini, and the statistical significance levels of the most important variables for COVID-19 mortality prediction calculated using XGBoost, Random Forest, and Chi-squared tests.

No	Features name	Chi-squared test		Random Forest	XGBoost	No	Features name	Chi-squared test		Random Forest	XGBoost
		Χ^2^	P-value	Mean decrease in Gini	Importance scores			Χ^2^	P-value	Mean decrease in Gini	Importance scores
1	Age	29.520	< 0.001	4.76	0.029	15	Absolute lymphocyte count	56.562	< 0.001	7.16	0.027
2	Sex	9.397	0.002	3.72	0.030	16	Absolute neutrophil count	10.290	0.006	2.37	0.017
3	Cough	23.077	< 0.001	4.19	0.026	17	Calcium	14.471	0.001	3.09	0.010
4	Underlying disease	20.450	< 0.001	2.33	0.015	18	Phosphor	11.612	0.003	3.98	0.008
5	Heart disease	20.921	< 0.001	3.36	0.022	19	Blood urea nitrogen	18.258	< 0.001	1.84	0.018
6	Hypertension	8.164	0.004	2.56	0.021	20	Total bilirubin	21.537	< 0.001	3.45	0.027
7	Diabetes	7.496	0.006	2.90	0.025	21	Serum albumin	15.818	< 0.001	3.16	0.017
8	Neurological disease	7.968	0.005	2.17	0.016	22	Glucose	15.005	0.001	4.58	0.016
9	Cancer	17.369	< 0.001	2.72	0.024	23	Creatinine kinase	27.031	< 0.001	5.15	0.023
10	Serum creatinine	55.795	< 0.001	7.11	0.053	24	Activated partial thromboplastic time	17.172	< 0.001	4.82	0.028
11	RBC	18.665	< 0.001	4.16	0.015	25	Prothrombin time	30.123	< 0.001	3.78	0.023
12	WBC	72.375	< 0.001	10.14	0.051	26	Hypersensitive troponin	22.836	< 0.001	2.49	0.022
13	Hematocrit	19.847	< 0.001	3.11	0.023	27	CTSS	71.482	< 0.001	11.22	0.048
14	Hemoglobin	23.882	< 0.001	3.94	0.022

**Table 4:** Descriptive statistics of the most important variables for mortality prediction in COVID-19 patients.

No	Features name	Variable type	Frequency or mean ± SD	No	Features name	Variable type	Frequency or mean ± SD
1	Age	Numeric	57.22 ± 16.76	15	Absolute lymphocyte count	Numeric	2.21 ± 1.17
2	Sex	Nominal	Female (368) Male (447)	16	Absolute neutrophil count	Numeric	7.37 ± 1.73
3	Cough	Nominal	Haven’t (209) Have (606)	17	Calcium	Numeric	9.52 ± 0.80
4	Underlying disease	Nominal	Haven’t (328) Have (487)	18	Phosphor	Numeric	3.48 ± 0.48
5	Heart disease	Nominal	Haven’t (648) Have (167)	19	Blood urea nitrogen	Numeric	39.69 ± 25.56
6	Hypertension	Nominal	Haven’t (571) Have (244)	20	Total bilirubin	Numeric	0.62 ± 0.50
7	Diabetes	Nominal	Haven’t (634) Have (181)	21	Serum albumin	Numeric	4.09 ± 0.46
8	Neurological disease	Nominal	Haven’t (776) Have (39)	22	Glucose	Numeric	137.45 ± 83.50
9	Cancer	Nominal	Haven’t (791) Have (24)	23	Creatinine kinase	Numeric	137.68 ± 234.68
10	Serum creatinine	Numeric	1.21 ± 0.56	24	Activated partial thromboplastic time	Numeric	28.26 ± 12.54
11	RBC	Numeric	4.58 ± 0.74	25	Prothrombin time	Numeric	12.76 ± 2.38
12	WBC	Numeric	7719.02 ± 3939.55	26	Hypersensitive troponin	Nominal	Normal (791) Abnormal (24)
13	Hematocrit	Numeric	39.61 ± 6.67	27	CTSS	Numeric	11.18 ± 5.28
14	Hemoglobin	Numeric	13.44 ± 2.15				

On the other hand, smoking^6,15,17,28,30^, alcohol/addiction^6,17,30^, sore throat^6,15–17,26,27,31,33,38^, myalgia and malaise^6,14–17,26,28,34^, diarrhea and gastrointestinal symptoms^6,14,16,17,29,30,36^, headache^6,11,17,26,30,31,37^, platelet count^6,14,28,29^, and alanine aminotransferase (ALT)^6,14,29,31^ were the irrelevant features in predicting COVID-19 mortality. Despite the clinical importance of these parameters for treatment success and mortality prediction, many of them could be eliminated from ML analysis and mortality prediction would be performed with fewer factors and the same accuracy.

### Evaluation of the developed models

In this study, COVID-19 mortality prediction models were developed using eight ML algorithms including J48, SVM, MLP, k-NN, NB, LR, RF, and XGBoost. These predictive models were built using the best feature subset determined in the previous step. The ML algorithms were trained using the same dataset. The performances of these models were evaluated using sensitivity, specificity, accuracy, precision, and AUC metrics. Results of the performance evaluation for the developed models are listed in Table 5.

**Table 5:** Performances of ML algorithms for mortality prediction in COVID-19 patients.

ML algorithms	Sensitivity (%)	Specificity (%)	Accuracy (%)	Precision (%)	F1-score	AUC
J48	98.4	84.9	91.7	86.7	92.2	93.9
NB	78.4	77.9	78.1	78	78.2	87
LR	81.5	79.9	80.7	80.2	80.8	88.9
MLP	98.4	91.1	94.8	91.7	94.9	97
SVM	83	79.3	81.2	80.1	81.5	81.2
k-NN	100	86.3	93.1	87.9	93.6	97.2
RF	100	94.5	97.2	94.8	97.3	99.9
XGBoost	100	91.1	95.6	92	95.8	98.2

J48: J48 Decision tree; NB: Naïve Bayes; LR: logistic regression; MLP: multi-layer perceptron; SVM: support vector machine; k-NN: k-nearest neighbourhood; RF: random forest; XGBoost: eXtreme gradient boosting.

**Figure 3:**
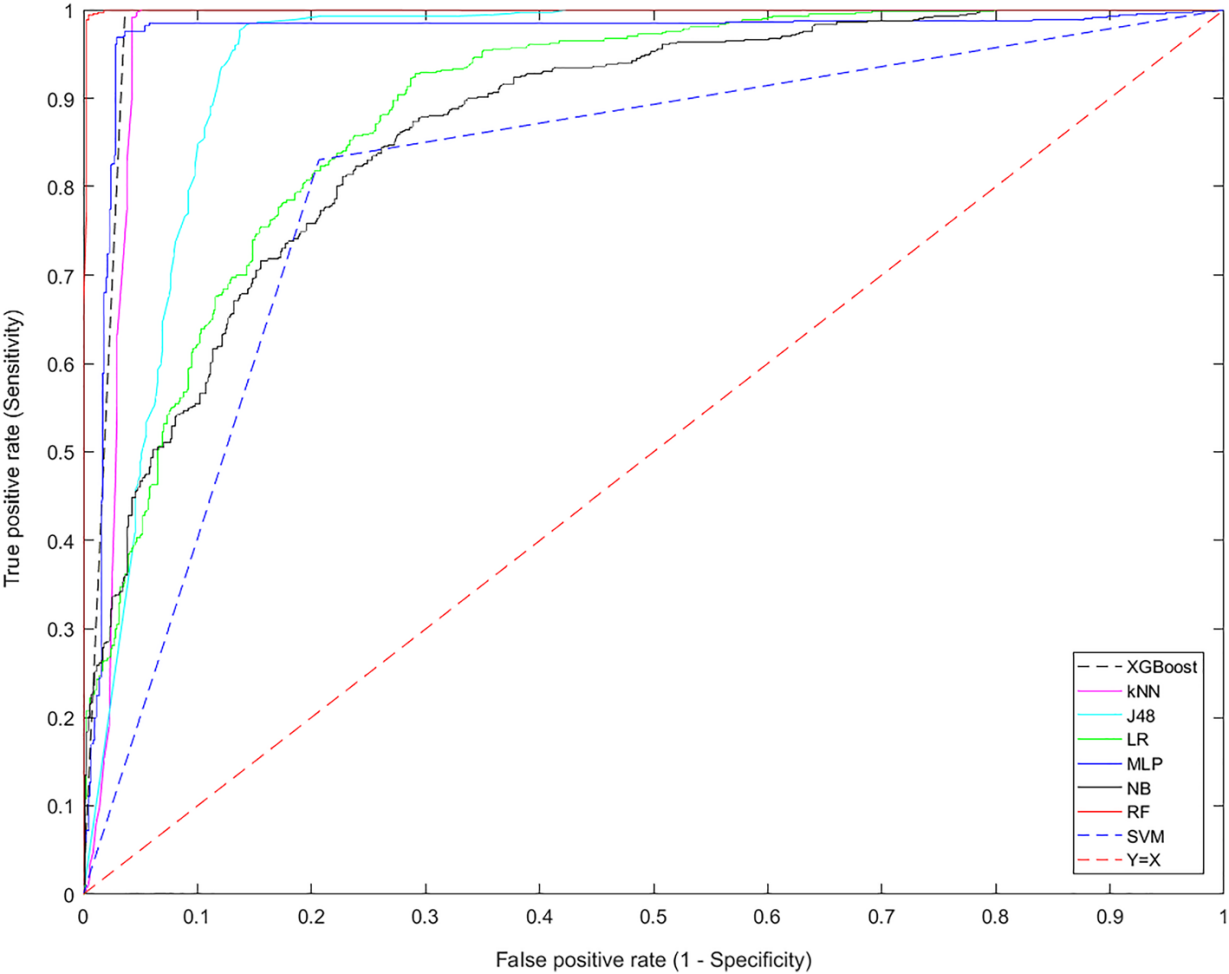
ROC curves for ML algorithms.

Results showed that the RF algorithm yielded better performance to predict the mortality of COVID-19 patients than other ML algorithms. The sensitivity, specificity, accuracy, precision, F1-score, and AUC of the RF algorithm were 100.0%, 94.5%, 97.2%, 94.8%, 97.3%, and 99.9%, respectively. Figure 3 depicted the comparison of the area under the ROC curve for the developed ML algorithms.

## Discussion

With the COVID-19 outbreak, the global health system had been faced a life-threatening infection with a wide range of symptoms and complications. For appropriate preparedness against to ongoing global pandemic, it is important to implement intelligent-based models for predicting which patients are at high risk for disease progression and poor outcomes. Timely and accurate identification of COVID‑19 patients with poor outcomes can guide physicians in selecting appropriate treatment and allocating limited hospital resources. AI has created remarkable opportunities to determine the best models for diagnosis, risk analysis, screening, and prediction in response to the challenges ahead of the healthcare system.

AI-based classification of chest scanning images for the automatic diagnosis of COVID-19 was evaluated by Jyoti et al. and Goel et al.^39,40^. The accuracy of MCA-inspired TQWT-based classification of chest X-ray images to the automatic diagnosis of COVID-19 was 98.82% and 94.64% for small and large datasets, respectively^39^. The AI system achieved an AUC of 0.92 to screen and detect COVID-19. Its diagnostic sensitivity is equal to a senior thoracic radiologist and for the patients with positive RT-PCR results and normal CT scans, the developed AI model improved the diagnosis of patients while the radiologist had reported them as COVID-19 negative^41^.

In Asteris et al. study^42^, AI approaches were used for early prediction of COVID-19 outcomes. They predicted intensive care unit (ICU) hospitalization of COVID-19 patients using artificial neural networks (ANN). Laboratory parameters of the adult patients were used to develop this predictive model. The accuracy, precision, sensitivity, and F1-score of the ANN for the validation cohort were 95.97%, 90.63%, 93.55%, and 92.06%, respectively. Their study showed that an AI-based predictive model could accurately predict ICU hospitalization using only 5 laboratory indices at the time of admission. These studies showed that AI could solve several issues affecting the diagnosis and prediction of COVID-19.

In the present study, we retrospectively analysed the data from a hospital-based registry database to develop and evaluate ML models capable of predicting the risk of COVID-19 mortality. First, demographic information, risk factors, clinical manifestations, laboratory results, and imaging findings were examined to identify the most relevant predictors for mortality prediction of COVID-19 patients. The selected set of the most relevant predictors was used to train and test ML algorithms. In our study, eight ML algorithms including the J48 decision tree, k-NN, MLP, SVM, XGBoost, NB, RF, and LR were used to develop the prediction models based on a dataset of laboratory-confirmed COVID-19 hospitalized patients. The results showed that RF with an accuracy of 97.2%, sensitivity of 100%, precision of 94.8%, specificity of 94.5%, F1-score of 97.3%, and AUC of 99.9% had the best performance among the other ML approaches. Decision tree, XGBoost, k-NN, and MLP models with AUC ≥ 93.9 showed good prediction performances in predicting COVID-19 mortality. Although other ML algorithms are categorized in the last ranks in terms of performance; they had also an acceptable performance (AUC ranged from 81.2 to 88.9%). The SVM model had the weakest performance among ML models (AUC = 81.2%).

The prognostic performances of ML techniques for the mortality prediction of COVID-19 patients have been evaluated in different studies. In Gao et al. study^14^, the mortality prediction of 2520 COVID-19 hospitalized patients was evaluated using LR, SVM, gradient boosted decision tree (GBDT), and neural network (NN) algorithms. For predicting COVID-19 patients’ physiological deterioration and death up to 20 days, the neural network-based prediction model with an AUC of 97.60% had a better performance than LR, SVM, and GBDT algorithms.

In Zakariaee et al. study^6^, the prognostic significance of chest CT severity score in mortality prediction of COVID-19 patients was evaluated using k-NN, MLP, SVM, and J48 decision tree ML approaches. The retrospective analysis of the data of 815 COVID-19 hospitalized patients showed that the prognostic performances of the ML algorithms would improve by the integration of CT-SS data with demographics, risk factors, clinical manifestations, and laboratory parameters. SVM was the weakest method in predicting mortality and the k-NN model with an accuracy of 94.1%, sensitivity of 100. 0%, precision of 89.5%, specificity of 88.3%, and AUC of around 97.2% had better performance than MLP, SVM, and J48 decision tree algorithms.

The prognostic performances of decision tree (J48), MLP, k-NN, random forest (RF), and SVM data mining models were also evaluated by Moulaei et al.^16^. The ML algorithms were developed using demographics, risk factors, and clinical manifestations of 850 COVID-19 hospitalized patients. Although all ML algorithms had good prognostic performances for mortality prediction of COVID-19 patients (AUCs > 96%), the RF model yields the best prognostic results and SVM was the weakest method.

In another study by Moulaei et al.^17^, the mortality prediction for 1500 COVID-19 hospitalized patients was performed using the decision tree (J48), RF, k-NN, MLP, Naïve Bayes (NB), eXtreme gradient boosting (XGBoost), and logistic regression (LR) algorithms. The results demonstrated that the RF model with an accuracy of 95.03%, sensitivity of 90.70%, precision of 94.23%, specificity of 95.10%, and AUC of 99.02 had the best performance. The results of these studies were in close agreement with our findings. A summary of these studies is presented in Table 6. In this table, developed ML models, datasets, and prognostic performances of ML models to predict mortality in COVID-19 patients are listed.

**Table 6:** The summary of studies describing ML models developed to predict mortality in COVID-19 patients.

ML algorithms	Datasets	Sensitivity (%)	Specificity (%)	Accuracy (%)	Precision (%)	F1-score	AUC
This study							
J48	28 predictors including patient’s demographics, clinical features, history of personal diseases/comorbidity, laboratory results, CT-SS, and output variable	98.4	84.9	91.7	86.7	92.2	93.9
NB		78.4	77.9	78.1	78	78.2	87
LR		81.5	79.9	80.7	80.2	80.8	88.9
MLP		98.4	91.1	94.8	91.7	94.9	97
SVM		83	79.3	81.2	80.1	81.5	81.2
k-NN		100	86.3	93.1	87.9	93.6	97.2
RF		100	94.5	97.2	94.8	97.3	99.9
XGBoost		100	91.1	95.6	92	95.8	98.2
Zakariaee et al.^6^							
J48	28 predictors including patient’s demographics, clinical features, comorbidity, laboratory results, and output variable	97.9	84.4	91.2	86.3	91.7	93.1
SVM		80.8	76.5	78.6	77.5	79.1	78.6
MLP		97.9	89.5	93.7	90.3	94.0	96.2
k-NN		100	87.0	93.5	88.5	93.9	97.5
Zakariaee et al.^6^							
J48	28 predictors including patient’s demographics, clinical features, comorbidity, laboratory results, CT-SS, and output variable	98.4	84.9	91.7	86.7	92.2	93.9
SVM		83.0	79.3	81.2	80.1	81.5	81.2
MLP		98.4	91.1	94.8	91.7	95.0	97.0
k-NN		100	88.3	94.1	89.5	94.5	97.2
Moulaei et al.^17^							
RF	39 predictors including demographics, risk factors, clinical manifestations, laboratory tests, therapeutic plans, and output variable	90.70	95.10	95.03	94.23	–	99.02
XGBoost		90.89	95.01	94.25	92.43	–	98.18
kNN		97.38	82.15	89.56	80.11	–	96.78
MLP		90.81	91.07	91.25	87.19	–	96.49
LR		91.45	84.47	91.23	83.94	–	94.22
J48		87.77	94.47	92.17	89.97	–	92.19
NB		90.44	84.31	87.47	81.32	–	92.05
Moulaei et al.^16^							
J48	17 predictors including demographics, risk factors, clinical manifestations, and output variable	98	97.38	97.61	95.84	–	98.0
MLP		95.25	98.76	97.42	97.94	–	98.9
kNN1, K = 1		95. 25	97.84	96.85	96.45	–	99.2
kNN2, K = 3		97.75	100	99.14	100	–	98.7
kNN3, K = 5		95	95.23	95.14	92.45	–	99.3
RF		98.25	99.84	99.23	99.74	–	100
SVM		98	96	96.47	93.73	–	96.6
Gao et al.^14^ Internal validation cohort (SFV)							
SVM	15 predictors including demographics, risk factors, clinical manifestations, and output variable	60.7	97.8	92.4	–	69.7	95.94
GBDT		60.7	96.6	91.5	–	69.6	94.54
LR		56.2	98.1	92.1	–	67.1	96.14
NN		51.7	98.9	92.1	–	65.3	96.15
Gao et al.^14^ External validation cohort (OV)							
SVM	15 predictors including demographics, risk factors, clinical manifestations, and output variable	50.0	99.5	95.8	–	63.8	97.74
GBDT		48.3	98.5	94.8	–	58.0	95.36
LR		45.0	99.5	95.4	–	59.3	97.21
NN		46.7	99.6	95.6	–	61.5	97.54
Gao et al.^14^ External validation cohort (CHWH)							
SVM	15 predictors including demographics, risk factors, clinical manifestations, and output variable	57.9	94.6	88.8	–	62.9	90.67
GBDT		31.6	99.0	87.9	–	46.2	90.21
LR		36.8	96.9	87.1	–	48.3	92.13
NN		47.4	96.9	88.8	–	58.1	92.02

In this table, developed ML models, datasets, and prognostic performances of ML models are listed.

J48: J48 decision tree; NB: Naïve Bayes; LR: logistic regression; MLP: multi-layer perceptron; SVM: Support vector machine; k-NN: k-nearest neighbourhood; RF: random forest; XGBoost: eXtreme gradient boosting; GBDT: gradient boosted decision tree.

These ML algorithms were mostly developed using demographics, risk factors, clinical manifestations, and laboratory parameters. Their dataset has no clinical imaging data. Chest CT is one of the most common methods used to evaluate and diagnose patients with suspected SARS-CoV-2 infection^41^. The systematic review and meta-analysis of chest CT manifestations in COVID-19 patients indicated that vascular enlargement, ground-glass opacities (GGOs), subpleural bands, and interlobular septal thickening were typical CT features of COVID-19 patients. These common patients are less likely to have radiographic abnormalities with over two lobes involved compared to severe patients. For severe patients, vascular enlargement, GGOs, interlobular septal thickening, air bronchogram, consolidation, subpleural bands, crazy-paving pattern, and traction bronchiectasis were the predominant CT features; and traction bronchiectasis, consolidation, interlobular septal thickening, crazy-paving pattern, reticulation, pleural effusion, and lymphadenopathy were related parameters to the severity of the disease^43^. The severity of pulmonary involvement on CT scans is significantly associated with mortality of COVID-19 patients (OR = 7.124 (95% CI 5.307–9.563)^19^ and it could predict the patient mortality with a sensitivity of 0.67 [95%CI (0.59–0.75)] and specificity of 0.79 [95%CI (0.74–0.84)]^1^.

In our study, the importance and efficiency of CT-SS to predict COVID-19 mortality were evaluated using three feature selection methods including XGBoost, random forest, and Chi-squared tests. Our results showed that CT-SS is one of the most important and relevant parameters to predict mortality risk in COVID-19 patients. A high importance score was observed for CT-SS in both XGBoost and random forest tests. In this study, similar to previous studies, deceased patients had higher CT-SSs and there was a significant statistical difference between the deceased and survived cases (P < 0.001). These findings indicate that CT-SS is a strong predictor to predict mortality risk in COVID-19 patients. Thus, the integration of this predictor with demographics, risk factors, clinical manifestations, and laboratory parameters, would improve prognostic performances of the ML algorithms for mortality prediction of COVID-19 patients.

These observations showed ML models are a valuable tool for making reliable clinical decisions and achieving evidence-based patient management to improve patient outcomes and the quality of medical care. The RF predictive models with the more comprehensive dataset including CT-SS could efficiently predict the mortality of COVID-19 patients. This could lead to the optimal use of hospital resources and an increased probability of survival of patients.

### Limitations

This study had some limitations. First, the predictive performances of the ML models to predict the mortality of COVID-19 patients were not evaluated in a prospective cohort due to the retrospective nature of the study. Second, this is a single-centre study and patients included were primarily local residents from Abadan, Iran. External validation of the proposed model merits future investigations on bigger and multi-centre databases.

## Conclusions

In this study, we compared the prognostic performances of the J48 decision tree, k-NN, MLP, SVM, XGBoost, NB, RF, and LR algorithms for mortality prediction of COVID-19 patients using a more comprehensive collection of features including CTSS data, demographics, risk factors, clinical manifestations, and laboratory findings. Results showed that timely and accurate risk stratification of COVID-19 patients could be performed using ML-based predictive models fed by routine data. The RF predictive model with a comprehensive collection of predictors could lead to promptly targeting high-risk patients on admission and therefore it would improve patient survival probability.

## Data Availability

The datasets used and/or analysed during the current study are available from the corresponding author on reasonable request.
